# Therapeutic dosages of aspirin counteract the IL-6 induced pro-tumorigenic effects by slowing down the ribosome biogenesis rate

**DOI:** 10.18632/oncotarget.11441

**Published:** 2016-08-20

**Authors:** Elisa Brighenti, Ferdinando Antonino Giannone, Francesca Fornari, Carmine Onofrillo, Marzia Govoni, Lorenzo Montanaro, Davide Treré, Massimo Derenzini

**Affiliations:** ^1^ Department of Experimental, Diagnostic and Specialty Medicine, University of Bologna, Bologna 40138, Italy; ^2^ Department of Medical and Surgical Sciences, University of Bologna, Bologna 40138, Italy; ^3^ Biomedical and Applied Research Center, University Hospital of Bologna, Policlinico S. Orsola-Malpighi, Bologna 40138, Italy

**Keywords:** aspirin, inflammation, cancer, ribosome biogenesis, p53

## Abstract

Chronic inflammation is a risk factor for the onset of cancer and the regular use of aspirin reduces the risk of cancer development. Here we showed that therapeutic dosages of aspirin counteract the pro-tumorigenic effects of the inflammatory cytokine interleukin(IL)-6 in cancer and non-cancer cell lines, and in mouse liver *in vivo*. We found that therapeutic dosages of aspirin prevented IL-6 from inducing the down-regulation of p53 expression and the acquisition of the epithelial mesenchymal transition (EMT) phenotypic changes in the cell lines. This was the result of a reduction in c-Myc mRNA transcription which was responsible for a down-regulation of the ribosomal protein S6 expression which, in turn, slowed down the rRNA maturation process, thus reducing the ribosome biogenesis rate. The perturbation of ribosome biogenesis hindered the Mdm2-mediated proteasomal degradation of p53, throughout the ribosomal protein-Mdm2-p53 pathway. P53 stabilization hindered the IL-6 induction of the EMT changes. The same effects were observed in livers from mice stimulated with IL-6 and treated with aspirin. It is worth noting that aspirin down-regulated ribosome biogenesis, stabilized p53 and up-regulated E-cadherin expression in unstimulated control cells also. In conclusion, these data showed that therapeutic dosages of aspirin increase the p53-mediated tumor-suppressor activity of the cells thus being in this way able to reduce the risk of cancer onset, either or not linked to chronic inflammatory processes.

## INTRODUCTION

A series of epidemiologic observations and population-based studies demonstrated that a regular use of the non-steroidal anti-inflammatory drug aspirin (acetylsalicylic acid) is associated with a reduced long-term risk for overall cancers [1–5], which is mainly due to a lower incidence of the gastrointestinal tract cancers, especially colorectal cancers [6].

Although this prevention effect may be not surprising, considering that there is a close relationship between inflammation and the development of many types of human cancers [7, 8], the mechanisms by which aspirin exerts its anticancer effects have not yet been clarified completely. Some mechanisms, either dependent on or independent of aspirin's specific inhibition of the cyclooxygenase, have been shown to be involved in reducing proliferation and angiogenesis, while enhancing cell differentiation and apoptotic death in tumor cells [1, 2, 9–13].

Inflammatory cells produce and release a series of factors which foster tumor initiation and promotion, two of the main steps in carcinogenesis [14, 15]. Among the various substances produced by the inflammatory cells, interleukin 6 (IL-6) appears to play a major role in tumorigenesis by stimulating proliferation and inhibiting apoptosis [16–21].

We recently highlighted an IL-6-related pathway that may connect inflammation to cancer: we found that IL-6 down-regulated the expression and activity of p53 in transformed and untransformed human cell lines, and that p53 down-regulation led to the acquisition of cellular phenotypic changes which are characteristic of epithelial-mesenchymal transition (EMT) [22]. P53 down-regulation was due to IL-6-dependent stimulation of c-Myc mRNA translation, which up-regulated rRNA transcription. The enhanced rRNA transcription reduced p53 expression through the activation of the well-established ribosomal protein (RP)-Mdm2 degradation pathway of p53 [23–26]. These changes occurred also in the colon epithelial cells of patients with ulcerative colitis -a typical example of chronic inflammation at high risk of tumor development [27]- and disappeared after the remission of the inflammatory process via long-term anti-inflammatory treatment with NSAID mesalazine (5-aminosalycylic acid) [22]. These observations prompted us to investigate whether pharmacological dosages of aspirin might neutralize the above-reported tumorigenic effects of IL-6 exposure. Here we demonstrated that in cancer and non-cancerous cell lines, as well as in mouse liver, therapeutic dosages of aspirin counteracted the increase in the ribosome biogenesis rate caused by IL-6, thus hindering p53 down-regulation with the consequent induction of EMT changes. These effects were observed to occur also in human cell lines and in mouse liver not stimulated by IL-6.

## RESULTS

### Aspirin, in a range of therapeutic dosages, does not cause apoptotic cell death

**Figure 1:**
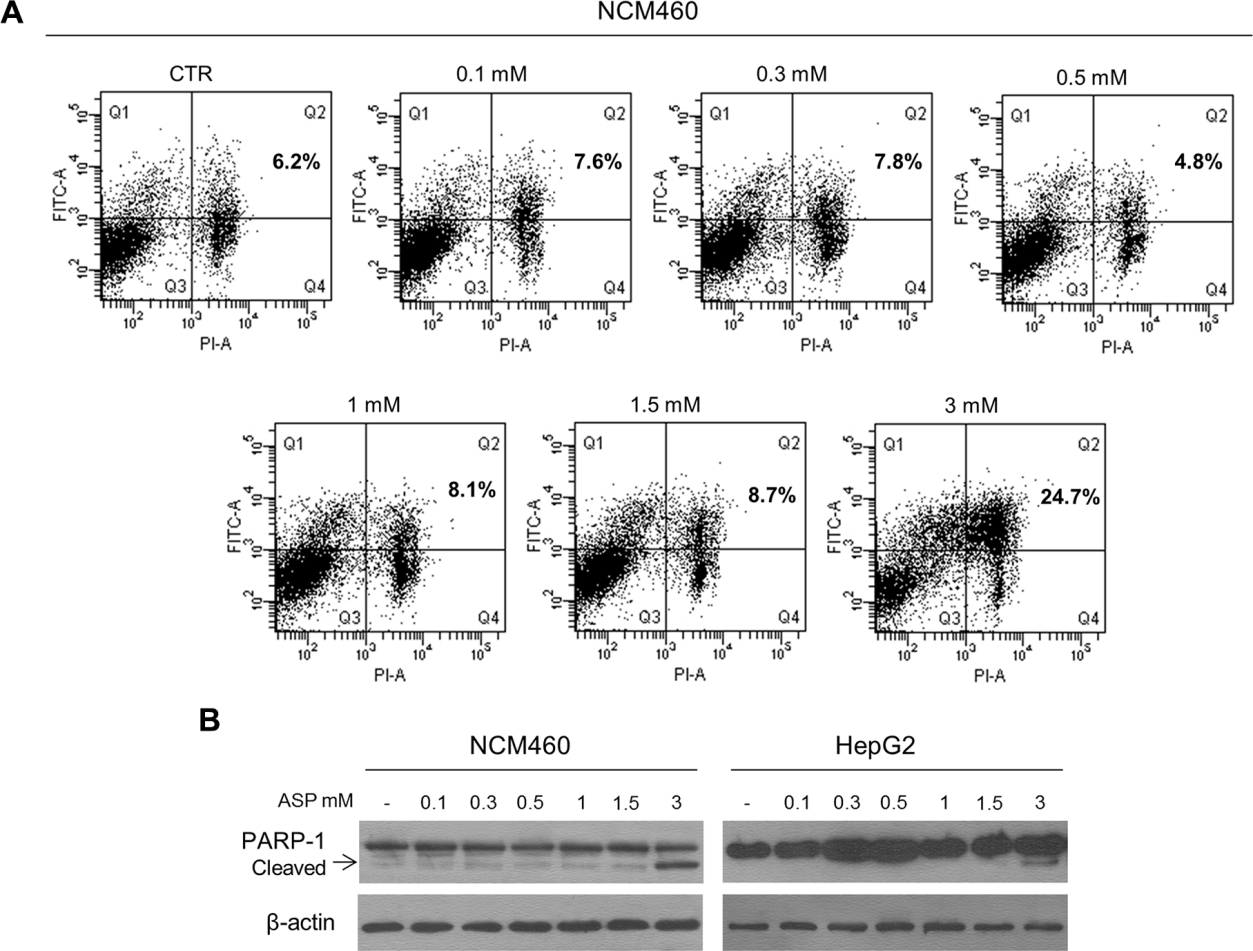
Aspirin, in the range of therapeutic dosages, does not cause apoptotic cell death (**A**) Representative flow cytometry DNA analysis for apoptosis detection using Annexin V–fluorescein isothiocyanate/propidium iodide double staining. NCM460 cells were treated with 0.1, 0.3, 0.5, 1, 1.5, 3 mM aspirin for 24 hours. Percentages of cells showing apoptosis are shown in the boxes (right upper quadrants). Only aspirin at a dosage of 3 mM induced the accumulation of apoptotic cells. (**B**) Representative Western blot of cleaved PARP-1 expression in NCM460 and HepG2 cells treated with 0.1, 0.3, 0.5, 1, 1.5, 3 mM aspirin for 24 h. The expression of cleaved PARP-1 is visible only in cells exposed to 3 mM aspirin.

Most studies on the anti-cancerous effect of aspirin have shown that the drug induced apoptotic death in cancer cells [28–33]. In most of these studies, however, apoptosis was induced by using much higher drug concentrations than those used for therapeutic purposes. For this reason, in a preliminary experiment we evaluated whether the concentrations of aspirin, in the therapeutic dosages range, can actually determine apoptotic cell death. In order to establish the apoptotic effect of aspirin, we evaluated both the number of Annexin V-positive cells by flow cytometry analysis [34] and the expression of cleaved PARP-1 by Western blot analysis [35] in two human epithelial cell lines: one from normal colon epithelium (NCM460 cell line) and one from hepatocellular carcinoma (HepG2 cell line). We found that aspirin, at concentrations ranging from 0.1 to 1.5 mM, did not induce significant changes in the number of apoptotic cells (Figure 1A) and in the expression of cleaved PARP-1 (Figure 1B) in both cell lines. Conversely, aspirin at the concentration of 3 mM was responsible for the increased number of apoptotic cells and PARP-1 cleavage. It is worth noting that while an aspirin concentration of 1.5 mM -as evaluated in the plasma of patients poisoned by acute aspirin overdose- is below the value associated with clinical signs of toxicity, this was not the case for the concentration of 3 mM, which was within the values associated with signs of acute toxicity [36]. For this reason, in the series of experiments conducted in order to investigate the mechanism by which aspirin may counteract the tumorigenic activity of IL-6, we used aspirin concentrations ranging from 0.1 mM to 1.5 mM.

### Aspirin counteracts the effect of IL-6 on c-Myc and p53 expression and on the induction of EMT

**Figure 2:**
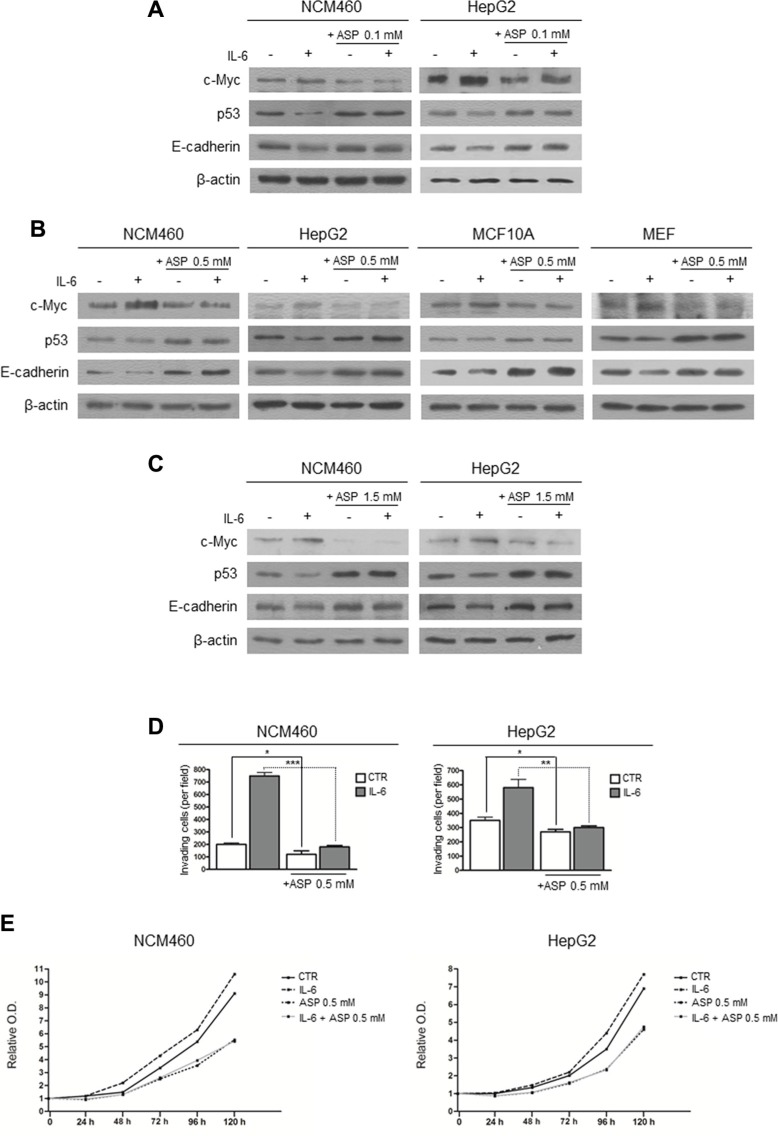
Aspirin counteracts the effect of IL-6 on c-Myc and p53 expression and on the induction of the EMT (**A**, **B**, **C**) Representative Western blot analysis of c-Myc, p53 and E-cadherin protein expression in control and IL-6-stimulated cells treated with aspirin for 24 hours. (A) NCM460 and HepG2 cells treated with aspirin at a concentration of 0.1 mM. (B) NCM460, HepG2, MCF10 and MEF cells treated with aspirin at a concentration of 0.5 mM. (C) NCM460 and HepG2 cells treated with aspirin at a concentration of 1.5 mM. In all cell lines, aspirin, at every dosage used, counteracts the effect of IL-6 on c-Myc, p53 and E-cadherin expression. Moreover, aspirin decreased c-Myc and increased p53 and E-cadherin expression, also in comparison with unstimulated control cells. (**D**) Invasion assay of NCM460 and HepG2 cells. Cells were exposed to IL-6 and/or to aspirin 0.5 mM for 24 hours. In both cell lines, aspirin counteracted the effect of IL-6 on the invasiveness potential. (**E**) Long-term effect (120 h) of aspirin at the dose of 0.5 mM on the proliferation rate in control and IL-6-stimulated NCM460 and HepG2 cells. In both cell lines, aspirin counteracted the effect of IL-6 on the cell proliferation rate. The histograms show the values (mean ± s.d.) of three experiments. *p*-value: *< 0.05; **< 0.01; ***< 0.001. O.D.: optical density.

Since IL-6 exposure increases the expression of c-Myc protein, down-regulates p53, and induces EMT [22], we wondered whether aspirin might counteract these IL-6-induced pro-tumorigenic effects in both cancer (HepG2) and non-cancerous (NCM460, MCF10A and MEF) cell lines. For this purpose, we treated IL-6-stimulated cells with aspirin at the concentrations of 0.1, 0.5, and 1.5 mM. As for the expression of c-Myc and p53, IL-6 treatment for 24 hours induced an increased c-Myc and a decreased p53 expression in all the cell lines used, in agreement with previously reported observations [22]. These effects were completely erased by a simultaneous exposure to either 0.1 mM (in NCM460 and HepG2 cells) or 0.5 mM (in NCM460, HepG2, MCF10A and MEF cells), or 1.5 mM aspirin (in NCM460 and HepG2 cells) (Figure 2A–2C). Using the same experimental conditions, we also evaluated the effect on E-cadherin expression, as its down-regulation represents a major phenotypic change in the EMT process [37, 38]. Aspirin completely counteracted the IL-6 down-regulating effect on E-cadherin expression (Figure 2A–2C). It is noteworthy that aspirin decreased c-Myc and increased p53 and E-cadherin expression, also in comparison with control, unstimulated cells (see Supplementary Figure S1 for densitometric analysis).

To further investigate the effect of aspirin on the EMT induction by IL-6, we evaluated the effect of IL-6 treatment on the invasiveness potential of NCM460 and HepG2 cells with or without aspirin at a concentration of 0.5 mM. Consistently with previous data [22, 39], we found that IL-6 enhanced the cell invasiveness and that this enhancement was erased by the simultaneous aspirin exposure (Figure 2D, and Supplementary Figure S2). Once again, aspirin reduced invasiveness also in comparison with unstimulated control cells.

We also evaluated the cell proliferation rate in NCM460 and HepG2 cells after IL-6 stimulation and aspirin treatment at a dosage of 0.5 mM. IL-6 was found to increase the proliferation rate in both cell lines in comparison with control cells. Aspirin reduced the proliferation rate to the same extent both in control and in IL-6-treated cells (Figure 2E).

Thus aspirin contrasted the activation of the EMT program, the increased invasiveness potential, and the stimulation of cell proliferation rate caused by IL-6 exposure.

### Therapeutic dosages of aspirin down-regulate c-Myc protein expression and slow down the ribosome biogenesis rate

**Figure 3:**
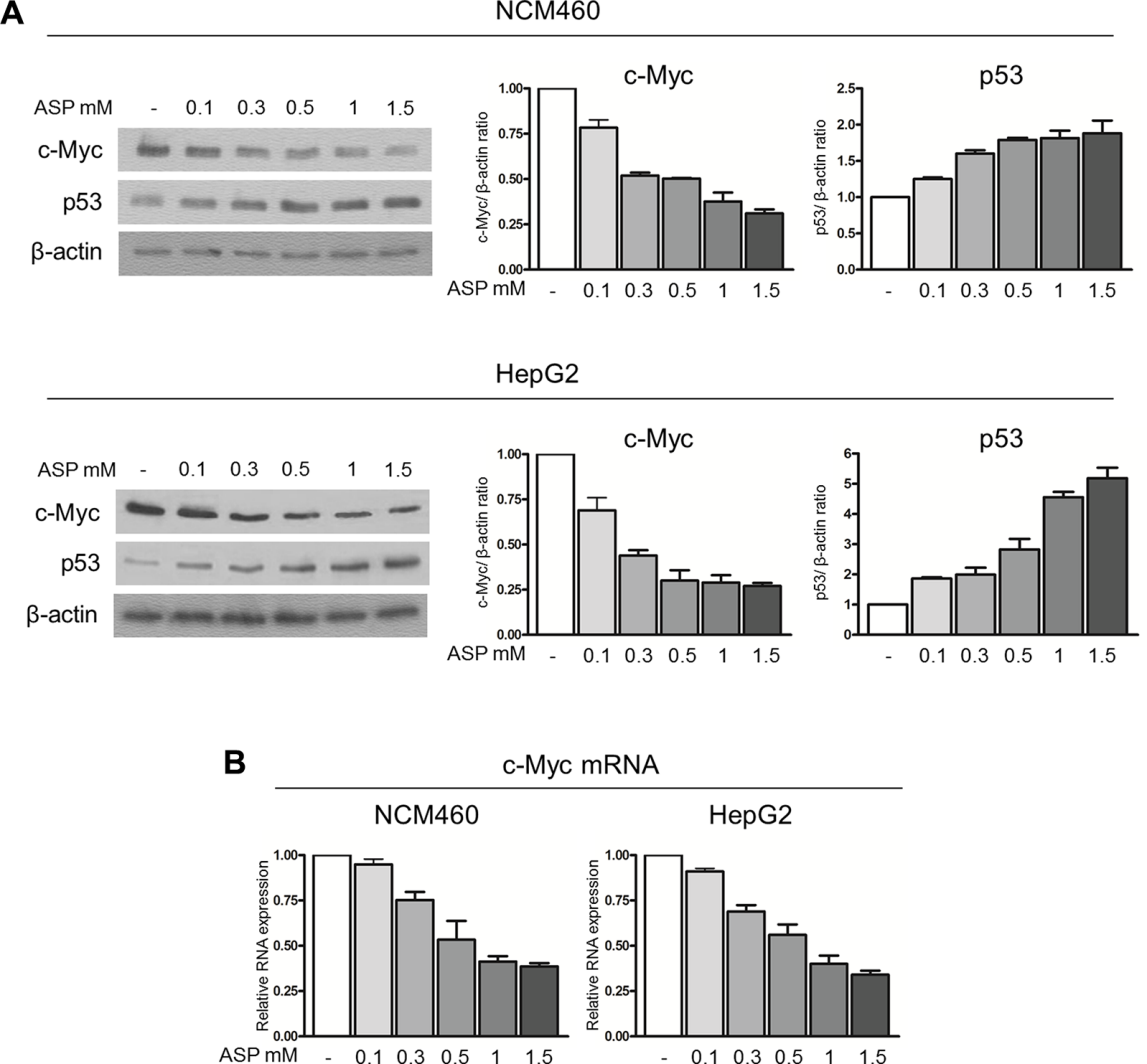
Therapeutic doses of aspirin down-regulate c-Myc protein and increase p53 expression (**A**) Representative Western blot and densitometric analysis of c-Myc and p53 protein expression in NCM460 and HepG2 cells treated with 0.1, 0.3, 0.5, 1, 1.5 mM aspirin for 24 h. A progressive decrease in c-Myc protein and a progressive increase in p53 protein expression occurred in both cell lines. (**B**) Real-time RT-PCR analysis of the c-Myc mRNA level in NCM460 and HepG2 cells exposed to 0.1, 0.3, 0.5, 1, 1.5 mM aspirin for 3 hours. The histograms show the values (mean ± s.d.) of three experiments.

In order to clarify the underlying mechanisms of aspirin action in counteracting the pro-tumorigenic effects of IL-6, we initially evaluated the effect of aspirin alone, at concentrations ranging from 0.1 to 1.5 mM, on c-Myc and p53 expression in NCM460 and HepG2 cells. We found that a progressive decrease in c-Myc protein and a progressive increase in p53 protein expression occurred in both cell lines treated for 24 hours with increasing aspirin concentration as evaluated by densitometric Western blot analysis (Figure 3A). Regarding the mechanism by which aspirin caused the c-Myc protein quantitative changes, we evaluated the early effects of an increasing aspirin concentration on c-Myc mRNA transcription. We found that a 3-hour treatment with increasing aspirin concentration progressively down-regulated c-Myc mRNA expression (Figure 3B), thus strongly suggesting that the decreased expression of c-Myc protein was a consequence of a reduced c-Myc mRNA level.

Regarding the mechanism leading to p53 stabilization, bearing in mind that c-Myc is the major modulator of ribosome biogenesis [40–42] and that perturbations of ribosome biogenesis stabilize p53 [23–26], we wondered whether the reduced c-Myc protein expression induced by aspirin might be responsible for a reduction in the ribosome biogenesis rate.

**Figure 4:**
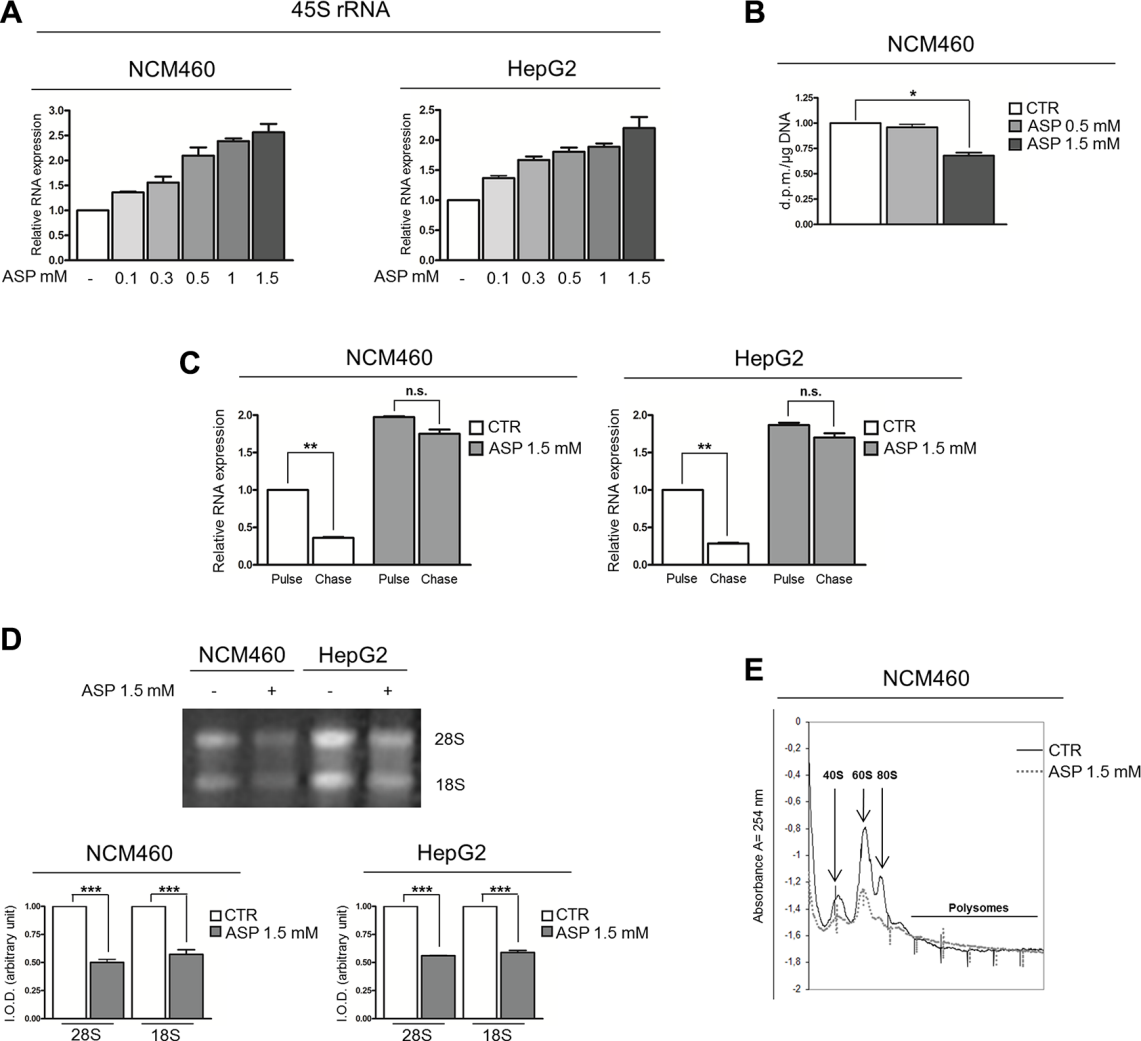
Therapeutic dosages of aspirin slow down the ribosome biogenesis rate (**A**) Real-time RT-PCR analysis of the 45S rRNA expression in NCM460 and HepG2 cells treated with 0.1, 0.3, 0.5, 1, 1.5 mM aspirin for 24 h. The increasing concentrations of aspirin caused a progressive increase in the amount of 45S rRNA. (**B**) RNA polymerase I activity assessment on NCM460 cells treated with 0.5 and 1.5 mM aspirin for 24h. No increase in RNA polymerase I activity was detected after 0.5 mM aspirin exposure; at a dosage of 1.5 mM, aspirin reduced rRNA transcription. (**C**) Analysis of 45S rRNA maturation in NCM460 and HepG2 cells treated with aspirin at a dosage of 1.5 mM for 24 h. Cells were labeled with 5-EU for 2 hours (pulse). The medium was then replaced with medium containing Uridine for 2 hours (chase). Real-time RT-PCR evaluation of 45S rRNA expression showed that, in control cells, the labeled 45S rRNA was greatly reduced after a 2-hour chase. In aspirin-treated cells, the amount of labeled 45S rRNA after a 2-hour pulse was higher than in control cells. However, after a 2-hour chase, no significant reduction in 45S rRNA was detectable. (**D**) 28S and 18S rRNA evaluation in NCM460 and HepG2 cells treated with 1.5 mM aspirin for 24 h. Total RNA from the same number of cells was size-separated on 1% agarose gel and stained with ethidium bromide. Two bands corresponding to 28S and 18S rRNA were visible in each lane. The quantitative analysis of 28S and 18S rRNA transcripts is also reported. IOD, integrated optical density. Aspirin reduced the amount of both 28S and 18S rRNA. (**E**) Polysome profile analysis of whole NCM460 extracts separated by sucrose gradient centrifugation. Samples were collected after 1.5 mM aspirin treatment for 24 hours. The 40S, 60S, 80S ribosome subunits and polysome fraction are indicated. The histograms show the values (mean ± s.d.) of three experiments. *p*-value: *< 0.05; **< 0.01; ***< 0.001. n.s., not significant.

For this purpose, we measured the rRNA level in NCM460 and HepG2 cells exposed for 24 hours to an increasing concentration of aspirin (from 0.1 to 1.5 mM) by Real-time RT-PCR analysis of 45S rRNA expression. For this purpose, we used a set of primers specific for the 5′-ETS region of the 47S/45 S rRNA transcript (see Supplementary Table S1). The results obtained were not consistent with the expected reduction in the ribosome biogenesis rate. In fact, a progressive increase in the amount of 45S rRNA in cells was noticed to occur at increasing concentration of aspirin (Figure 4A). This effect was also recorded in the cells stimulated by IL-6, in which aspirin further increased the 45S rRNA amount (Supplementary Figure S3).

To further investigate the effect of aspirin treatment on the ribosome biogenesis, we measured the RNA polymerase I activity on NCM460 cells treated with 0.5 and 1.5 mM aspirin. This methodological approach failed to reveal any increase in rRNA synthesis, which was even reduced at the higher aspirin concentration used (Figure 4B). The latter observation indicated that the increased amount of 45S rRNA detected after aspirin treatment by Real-time RT-PCR analysis cannot be considered the result of an increased rRNA transcriptional activity, but rather suggests that an altered rRNA maturation process might be responsible for the 45S rRNA accumulation. For this reason, we measured the rRNA maturation rate in NCM460 and HepG2 cells after aspirin exposure, using a non-radioactive labeling approach. In this experiment we used the highest, non-toxic concentration of aspirin (1.5 mM) in order to obtain more clear-cut results. We labeled NCM460 and HepG2 cells with 5-EthynylUridine (EU), for 2 hours. After a chase time of 4 hours with Uridine, RNA was extracted and the labeled RNA bound to a Biotin-Azide compound. At the end of the reaction the nascent RNA was captured by Streptavidin beads. We then performed a Real-time RT-PCR analysis on the captured RNA, in order to evaluate the expression of newly synthesized 45S rRNA. The data obtained showed that, in control cells after 4 hours of chase time, the newly synthesized 45S rRNA level was reduced, whereas in aspirin-treated cells the newly synthesized 45S rRNA level showed no significant change (Figure 4C). Accordingly, aspirin reduced the amount of 28S and 18S rRNA in NCM460 and HepG2 cells in comparison with control cells (Figure 4D), thus indicating that aspirin was responsible for a reduced maturation rate of newly synthesized 45S rRNA. Moreover, we found that aspirin-even at the 0.5 mM dosage-neutralized the increased production of 28S and 18S rRNA induced by IL-6 stimulation (Supplementary Figure S4).

The analysis of the polysome profile revealed that aspirin caused a down-regulation of the production of both 40S and 60S subunits, as well as of 80S mature ribosome particles, thus indicating that aspirin actually reduced the rate of ribosome biogenesis (Figure 4E).

**Figure 5:**
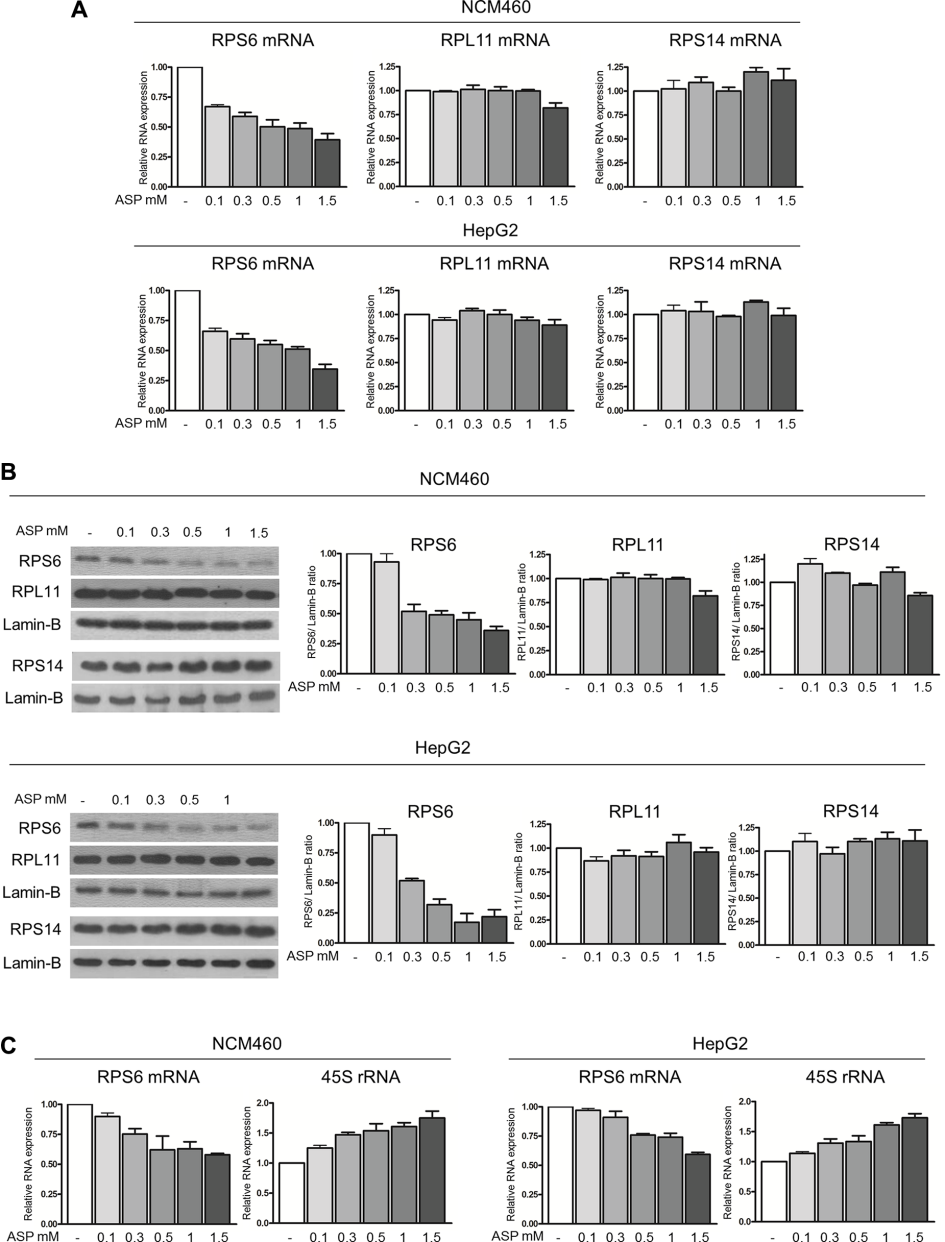
Therapeutic doses of aspirin down-regulate RPS6 mRNA and protein expression (**A**) Real-time RT-PCR analysis of the RPS6, RPL11 and RPS14 mRNA levels in NCM460 and HepG2 after treatment with 0.1, 0.3, 0.5, 1, 1.5 mM aspirin for 24 h and (**B**) representative Western blot and densitometric analysis of RPS6, RPL11 and RPS14 protein expression in NCM460 and HepG2 cells treated with 0.1, 0.3, 0.5, 1, 1.5 mM aspirin for 24 h. The increasing concentrations of aspirin caused a progressive reduction in the RPS6 mRNA and protein expression, whereas neither RPL11 and RPS14 mRNA nor RPL11 and RPS14 protein expression was reduced after aspirin exposure. (**C**) Real-time RT-PCR analysis of the RPS6 mRNA and of the 45S rRNA levels in NCM460 and HepG2 cells treated with 0.1, 0.3, 0.5, 1, 1.5 mM aspirin for 3 h. Then histograms show the values (mean ± s.d.) of three experiments.

In order to investigate the mechanistic relationship between the c-Myc protein down-regulation and the slow-down of the ribosome biogenesis rate, we took into consideration that c-Myc protein, in addition to stimulating the transcription of the rRNA precursor, is also necessary for its efficient processing [43]. The altered regulation of rRNA processing is very likely the result of the fact that c-Myc protein expression is directly related to the level of RPS6 expression [44]. Moreover, RPS6 depletion by siRNA causes a stop in rRNA maturation with accumulation of 45S rRNA [45]. For this reason, we first evaluated the effect of c-Myc mRNA interference on RPS6 mRNA expression in control and IL-6 stimulated NCM460 cells. We found that IL-6 highly stimulated RPS6 mRNA expression and that c-Myc mRNA silencing reduced the expression of RPS6 mRNA both in IL-6 exposed and in control cells (Supplementary Figure S5). We then measured the expression of RPS6 mRNA in aspirin-treated NCM460 and HepG2 cells. Real-time RT-PCR analysis indicated a reduced expression of RPS6 mRNA after 24-hour aspirin exposure, together with a progressive reduction in the amount of RPS6 protein, as evaluated by densitometric Western blot analysis, by increasing (from 0.1 to 1.5 mM) the concentrations of aspirin (Figures 5A and 5B). In order to rule out the possibility that the RPS6 protein reduction might be due to a general, non-specific ribosomal protein down-regulation, we evaluated the expression of two ribosomal proteins: one belonging to the large ribosome subunit (RPL11) and the other one to the small ribosome subunit (RPS14) after aspirin treatment in NCM460 and HepG2 cells. Neither the mRNA nor the protein expression of both proteins appeared to be reduced after aspirin exposure (Figure 5A and 5B). The reduction in RPS6 mRNA was a very early event, being detectable after a 3-hour aspirin treatment both in NCM460 and HepG2 cells (Figure 5C). Interestingly, this reduction was associated with a progressive increase in 45S rRNA amount as evaluated 3 hours after treatment with increasing (from 0.1 to 1.5 mM) concentrations of aspirin (Figure 5C).

Altogether, these data indicated that aspirin slowed down the ribosome biogenesis rate through the c-Myc/RPS6 pathway.

### Aspirin increases p53 protein expression through the RP/Mdm2/p53 pathway

**Figure 6:**
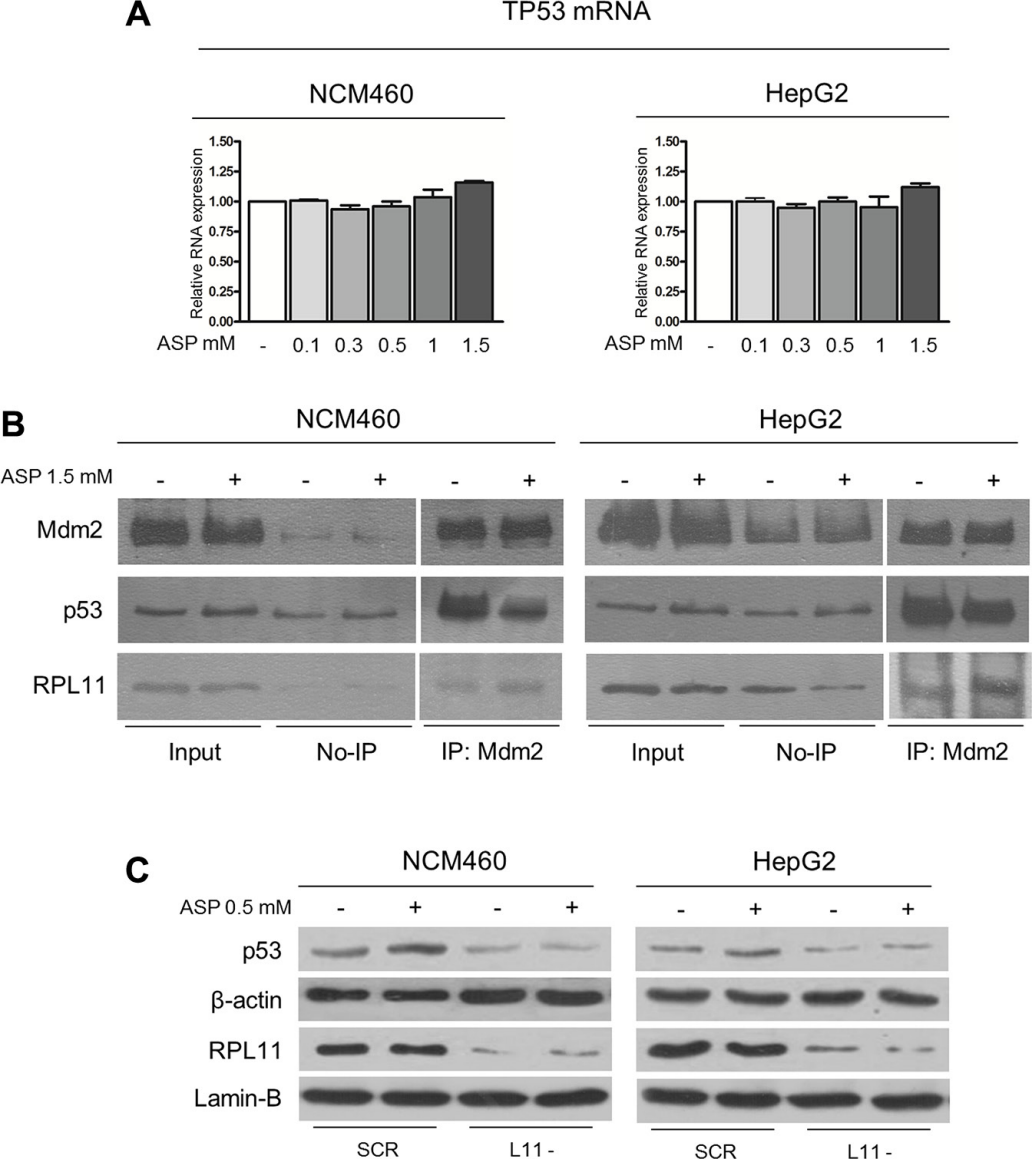
Aspirin increases p53 protein expression through the RP/Mdm2/p53 pathway (**A**) Real-time RT-PCR analysis of the TP53 mRNA expression in NCM460 and HepG2 cells treated with 0.1, 0.3, 0.5, 1, 1.5 mM aspirin for 24 h. (**B**) Co-immunoprecipitation of the amount of p53 and RPL11 protein bound to Mdm2 in control and aspirin-treated NCM460 and HepG2 cells. Cells were treated with aspirin at a dosage of 1.5 mM for 24 h. The amount of Mdm2, p53, and RPL11 is shown in the first two lanes before immunoprecipitation (input). The third and fourth lanes show the quantity of non-immunoprecipitated proteins (No-IP). The fifth and sixth lanes show the amount of Mdm2, p53, and RPL11 proteins after immunoprecipitation with anti-Mdm2 polyclonal antibody (IP: Mdm2). The histograms show the values (mean ± s.d.) of three experiments. (**C**) Western blot analysis of RPL11and p53 protein expression in NCM460 and HepG2 cells transfected with control sequences (SCR) and RPL11 siRNA1 (L11-). 48 h after the end of the silencing procedure, cells were exposed to 0.5 mM aspirin for 24 h. RPL11 silencing caused a reduction of RPL11 protein expression in both cell lines. Aspirin induced a p53 stabilization only in control cells. **P < 0.01. The same results were obtained using RPL11 siRNA2 (data not shown).

With regard to the mechanism involved in the p53 stabilization by aspirin, we wondered whether the slow-down of ribosome biogenesis rate may down-regulate p53 proteasomal degradation through the RPs/Mdm2 pathway. In fact, there is evidence that disturbed rRNA transcription allows ribosomal proteins, no longer used for ribosome building, to bind to Mdm2, thus reducing the Mdm2-mediated proteasomal degradation of p53 with consequent p53 stabilization [23–26]. Since it has been shown that aspirin, at very high concentration (5 mM) reduced the phosphorylation of STAT3 [32] and that activated STAT3 binds to the TP53 gene promoter repressing the transcription of TP53 mRNA [46], we first evaluated the transcription level of TP53 mRNA in the NCM460 and HepG2 cells by Real-time RT-PCR analysis after exposure to progressive concentrations of aspirin. The results obtained showed no significant difference between control and aspirin-treated cells (Figure 6A), thus indicating that in our experimental conditions the aspirin-induced p53 accumulation was not due to an increased TP53 mRNA transcription. On the other hand, the increased p53 stabilization appeared to be the result of an increased ribosomal protein binding to Mdm2, which reduced the capability of Mdm2 to bind p53 for digestion. In fact, the co-immunoprecipitation analysis showed that the quantity of RPL11 co-immunoprecipitated with Mdm2 was increased in aspirin-treated cells as compared to control cells, whereas the amount of p53 was reduced (Figure 6B).

To rule out the possibility that aspirin might mediate the effect on p53 independently of a reduction in ribosomal protein availability for Mdm2 binding, we down-regulated the expression of RPL11 mRNA by the small-interfering RNA procedure and evaluated the effect of aspirin treatment on p53 expression in NCM460 and HepG2 cells (Supplementary Figure S6 for siRNAs' validation). RPL11 mRNA silencing reduced RPL11 protein expression in both cell lines and hindered p53 stabilization after aspirin treatment (Figure 6C). This observation confirmed the importance of the increased ribosomal proteins availability for Mdm2 binding, in particular of RPL11, in the aspirin-induced p53 stabilization.

Considering that p53 controls the G1/S phase transition, we evaluated the distribution of NCM460 cells in the cell cycle phases after the treatment with increasing aspirin concentrations. We found that aspirin induced a mild accumulation of cells in the G1 phase, which was directly related to the drug concentration values (Supplementary Figure S7). This is in agreement with the observation that an increasing concentration of aspirin induced a progressive reduction in the cell proliferation rate (Supplementary Figure S8).

### Aspirin counteracts the pro-tumorigenic effects of IL-6 in mouse liver *in vivo*

We investigated whether aspirin may counteract those effects of IL-6 stimulation which are considered to facilitate the onset of tumors also in *in vivo* experimental conditions. For this reason, we treated mice with IL-6 alone (40 μg/kg), with aspirin alone (30 mg/Kg), and with IL-6 and aspirin simultaneously. The treatment with either IL-6 or aspirin or with both substances was given at time 0 and 6 hours later; mice were sacrificed 12 hours after the first treatment. Livers from 6 mice for every set of experimental conditions and for control were used.

**Figure 7:**
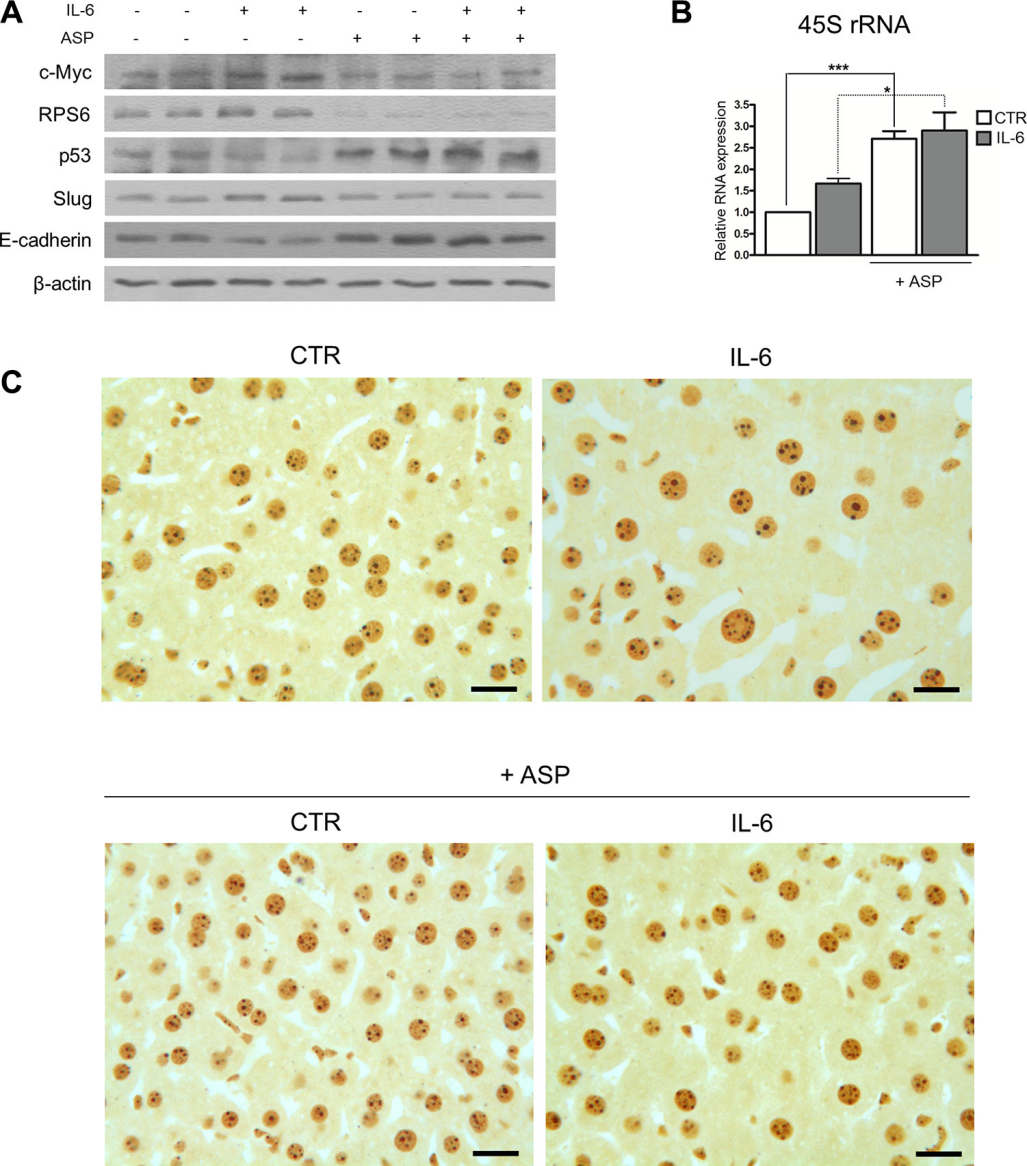
Aspirin counteracts the pro-tumorigenic effects of IL-6 in mouse hepatocytes *in vivo* (**A**, **B**, **C**) For *in vivo* experiments, mice were intraperitoneally injected with IL-6 at a dosage of 40 μg/kg and/or treated with aspirin at a dosage of 30 mg/Kg. Two doses of both substances were injected: at time 0 h and time 6 h. Mice were sacrificed 12 hours after the beginning of the experiment. (A) Representative Western blot analysis of c-Myc, RPS6, p53, Slug and E-cadherin expression from liver samples of C57BL/6 mice. Aspirin neutralized the quantitative changes induced by IL-6 on the expression of the proteins considered. (B) Real-time RT-PCR analysis of the 47S rRNA expression. Note the increased amount of 45S rRNA after IL-6 treatment and its further increase in aspirin-treated mice. *p*-value: *< 0.05; ***< 0.001. (C) Histological sections from liver samples stained following the silver procedure for the selective visualization of the argyrophilic AgNOR proteins. The area occupied by the silver-stained nucleolar structures was greater in IL-6-stimulated hepatocytes than in control hepatocytes. Aspirin significantly reduced the quantity of the silver-stained nucleolar structures in both IL-6 stimulated and control hepatocytes. Scale bar: 15 μm.

We found that IL-6 induced an increase in c-Myc protein expression, as measured by Western blot analysis, in comparison with control livers (Figure 7A). Aspirin completely hindered the rise of the amount of c-Myc protein. We also found that aspirin reduced the RPS6 protein expression in both control and IL-6-stimulated mice. As for p53, its amount was reduced after an IL-6 injection, as compared to controls, whereas it was increased after aspirin treatment in both controls and IL-6-stimulated mice (Figure 7A). We then analyzed whether IL-6 induced in mouse liver those phenotypical changes which are characteristic of EMT and whether aspirin might counteract these changes. For this purpose, we evaluated the expression of both the transcription factor Slug, responsible for EMT by repressing E-cadherin expression [47], and of E-cadherin itself. Slug expression was increased in IL-6-treated mice as compared to control mice; this increase was hindered by aspirin. As expected, the quantitative variations of E-cadherin expression were the opposite of those of Slug: in fact, E-cadherin expression was reduced in mice treated with IL-6 alone, whereas it appeared to be increased after aspirin treatment. Aspirin induced an increase in E-cadherin expression also in comparison with unstimulated control livers (Figure 7A).

Regarding the ribosome biogenesis rate, we observed that IL-6 treatment increased the amount of 45S rRNA as evaluated by Real-time RT-PCR analysis, in agreement with previous findings, showing that IL-6 stimulates rRNA transcription in human cell lines [22]. On the other hand, aspirin treatment increased the amount of 45S rRNA in comparison with both control and IL-6 stimulated mouse livers (Figure 7B). As in cell lines, this increase, as evaluated by Real-time RT-PCR analysis, was not confirmed when using another procedure to evaluate the ribosome rate: the measurement of the size of nucleoli in histological sections. In fact, when using selective silver staining for the argyrophilic nucleolar organizer region (AgNOR) proteins, it is possible to precisely evaluate the nucleolar size [48, 49]. Since the area occupied by nucleoli within the cell nucleus is closely and directly related to rRNA transcription activity [50], we were able to obtain information on changes in the ribosome biogenesis rate recorded in hepatocytes after IL-6 stimulation and aspirin treatment. Morphometric analysis and statistical evaluation indicated that the area of silver-stained nucleolar structures per hepatocyte nucleus from IL-6 treated mice was significantly larger than that from control mice (6.78 μm ± 2.29 SD *vs* 4.13 μm ± 1.66 SD; *p* < 0.001) (Figure 7C), thus showing that IL-6 actually stimulated rRNA transcription. On the other hand, aspirin by itself did not induce an increase in the area of silver-stained nucleolar structures per hepatocyte nucleus which, instead, appeared to be significantly reduced in comparison with control hepatocytes (*p* = 0.022), thus indicating that, *in vivo* also, aspirin did not stimulate, but in fact reduced rRNA transcription. As expected, aspirin treatment erased the increase in the area of nucleoli per nucleus in hepatocytes stimulated by IL-6, the value after aspirin treatment being quite similar to that of unstimulated control hepatocytes (3.86 μm ± 1.38 SD *vs* 4.13 μm ± 1.66 SD; *p* = 0.257 NS) (Figure 7C).

Taken all together, these data indicated that therapeutic dosages of aspirin not only neutralized the pro-tumorigenic effects of IL-6 stimulation in mouse hepatocytes *in vivo*, but also increased the p53-related anti-tumor potential in unstimulated control hepatocytes.

## DISCUSSION

The present data demonstrated that therapeutic dosages of aspirin counteract the down-regulation of p53 expression and the major EMT changes, i.e. the reduction in E-cadherin expression and increased cell invasiveness [51, 52], which are induced by IL-6 stimulation in cancer and non-cancerous cell lines. The counteracting activity of aspirin on the IL-6 induced pro-tumorigenic effects was also demonstrated *in vivo* in mouse liver.

In a previous study we showed that IL-6 increased c-Myc expression, which in turn enhanced ribosome biogenesis, resulting in a reduced availability of ribosomal protein to bind Mdm2, thus increasing the Mdm2-mediated digestion of p53. The down-regulation of p53 expression was responsible for the induction of EMT changes [22]. Here we showed that aspirin reduced the transcription of c-Myc mRNA, slowed down the rRNA maturation rate, and reduced the Mdm2-mediated digestion of p53. The reduction in c-Myc mRNA transcription was a very early event, that, in our experimental conditions, appeared to have a minor effect on rRNA transcription, whereas it exerted a slowing-down effect on the process of rRNA maturation, thus reducing the ribosome biogenesis rate. The latter effect is in agreement with the finding that c-Myc not only controls the RNA-polymerase I activity, but also directly affects the rRNA maturation rate [43]. The effect on ribosome biogenesis was very likely the result of a reduced expression of RPS6 protein, which we found to progressively decrease as aspirin concentration increased. In fact, c-Myc protein binds to the RPS6 promoter site [44] and the quantitative mRNA analysis demonstrated that c-Myc directly affected the expression level of RPS6 mRNA [53 and present results]. The perturbation of ribosome biogenesis caused by aspirin was responsible for the reduction of the Mdm2-mediated proteasomal digestion of p53 throughout the RPs-Mdm2-p53 pathway [23–26]. The neutralization of the IL-6-induced p53 protein down-regulation caused by aspirin was accompanied by a lack of EMT changes, which were instead a characteristic consequence of interleukin stimulation. These protective effects against the pro-tumorigenic action of IL-6 were recorded when using aspirin even at a very low (0.1 mM) concentration.

The observation that treatment with two other NSAIDs, sodium salicylate and mesalazine (5-aminosalicylic acid) which lack acetyl groups, increased p53 expression in control and IL-6 stimulated cell lines, as revealed by Western blot analysis, indicated that the above reported effects of aspirin were not due to unspecific acetylation-linked mechanisms. Moreover, as well as aspirin, these drugs increased the level of 45S rRNA as evaluated by Real-time RT-PCR analysis (Supplementary Figure S9).

The present study suggests that the mechanism by which aspirin and other NSAIDs exert an anti-tumor activity in the inflammatory chronic conditions was based on the neutralization of the p53 down-regulation and the consequent EMT activation, both induced by IL-6. A similar mechanism may be at the basis of the protective effect of mesalazine treatment [54] against colorectal cancer development in patients with ulcerative colitis, a chronic inflammation of the colon which is associated with an important risk for the onset of colorectal cancer [55–57].

Indeed, in a previous study conducted using colon biopsy samples we found an upregulation of ribosome biogenesis, a reduced expression of p53, together with a focal reduction or absence of E-cadherin expression in the colon epithelium from patients with ulcerative colitis, in comparison with normal mucosa samples. All these pro-tumorigenic changes disappeared after treatment with mesalazine [22].

It is worth noting that our results demonstrated that aspirin not only neutralized the pro-tumorigenic effects of IL-6 stimulation, but also stabilized p53, and hindered EMT activation in unstimulated control cells, thus suggesting that the effects of aspirin exposure were independent of IL-6 signalling. The independence of the aspirin effects from IL-6 signalling was demonstrated by the observation that silencing the expression of IL-6 mRNA did not hinder either p53 stabilization or 45S rRNA accumulation in HepG2 cells exposed to the drug (Supplementary Figure S10).

May this effect of aspirin be responsible for an anti-tumor surveillance in normally healthy people? That this can be the case is strongly suggested by the observation that regular aspirin users, in comparison with non-users, had a statistically significant reduced risk for cancers of the colon and rectum, and a reduced risk, even though not significant, for gastroesophageal cancers [6]. In fact, the aspirin doses as those generally taken by long-term users for vascular disease prevention (75–100 mg daily), may reach within the intestinal lumen concentrations which we have found to stabilize p53 in cell lines (e. g. 75–100 mg/L correspond to 0.41–0.55 mM). Interestingly, the regular use of aspirin was not associated with a reduced risk for non-gastrointestinal tract cancers, such as lung, breast and prostate [6]. In fact, after absorption and dilution in the body fluids the drug concentration which comes in contact with the other tissues is strongly reduced and very likely insufficient to induce p53 stabilization.

The anti-tumor activity of aspirin may be exerted other than through the well-established tumor-suppressor activity of p53, also by the down-regulation of ribosome biogenesis rate which, in experimental models, has been shown to reduce per se the development of spontaneous tumors [58, 59]. In this context, aspirin may help in preventing colorectal cancer onset also in those conditions in which a hyperinsulinemic status (another pathological condition associated with an increased risk of developing colorectal cancer [60, 61],) with the activation of insulin and IGF-1 pathways [62, 63], may be per se responsible for the stimulation of rRNA transcription and p53 down-regulation [64]. Accordingly, we observed that 0.5 mM aspirin neutralized the down-regulation of p53 and E-cadherin expression, both induced by IGF-1 in MCF-7 cells (data not shown).

The present results may explain also why a continued long-term use of aspirin was required for an anticancer effect-up to 15 or 20 years- before a reduced risk of colorectal cancer is recorded, whereas there was no protective effect after discontinuing its use [65]. In fact, aspirin, by reducing the ribosome biogenesis rate and increasing p53 expression slightly, induces a moderately greater resistance to carcinogenic insults which must be extended for a long period of time in order to obtain statistically significant effects in cancer prevention.

## MATERIALS AND METHODS

### Cell culture and chemical treatments

HepG2 and MCF10A were obtained from ATCC between 2005 and 2009. The NCM460 cell line was purchased from INCELL Corporation (San Antonio, TX, USA) in 2014. All the cell lines were p53 wild-type. C57BL/6JOlaHsd mice were used for primary mouse embryonic fibroblast (MEF) isolation. Recombinant human IL-6 (Sigma-Aldrich, Milan, Italy) was used at a final concentration of 50 ng/ml; mouse IL-6 (Sigma-Aldrich) was used at a final concentration of 40 μg/kg; aspirin (acetylsalicylic acid, Sigma-Aldrich) was used at 0.1, 0.3, 0.5, 1, 1.5 and 3 mM in cell lines, at 30 mg/kg in mice; salicylate (Sigma-Aldrich) was used at 0.5 mM; mesalazine (5-ASA, Sigma-Aldrich) was used at 1 mM.

### Animals

24 C57BL/6JOlaHsd mice (male, weighing 20–25 g) were purchased from Harlan Laboratories (Udine, Italy) at 7 weeks of age. Mice had access to food and water ad libitum. All procedures were conducted according to the guidelines for care.

Mice were randomly divided into four experimental groups:

- 6 mice were intraperitoneally injected with IL-6 at the dose of 40 μg/kg. Two doses were injected: at time 0 h and time 6 h.
- 6 mice were treated with acetylsalicylic acid by oral gavage at the dose of 30 mg/kg. Two doses were injected: at time 0 h and time 6 h.
- 6 mice were intraperitoneally injected with IL-6 at the dose of 40 μg/kg (two doses at time 0 h and time 6 h) and treated with acetylsalicylic acid at the dose of 30 mg/kg by oral gavage (two doses at time 0 h and time 6 h).
- 6 mice were intraperitoneally injected and treated by oral gavage with saline and used as controls.

Mice were sacrificed at 12 h and liver samples were collected. Experimental procedures were approved by the Ethical Committee of Bologna University.

### Annexin V assays

Detection of apoptotic cells was performed by using the Annexin V/Propidium Iodide detection kit (Bender MedSystems, Vienna, Austria) according to the manufacturer's instructions and analyzed by FACS.

### Immunoblotting and immunoprecipitation

The immunoblotting, isolation of nuclear protein fractions, immunoprecipitation and mice protein extraction procedures were performed as previously described [22].

The primary antibodies used in this work are as follows: human anti-p53 (clone BP53-12, Novocastra Laboratories, Newcastle Upon Tyne, UK), mouse anti-p53 (clone 1C12, Cell Signaling Technology, Beverly, MA, USA), anti-RPL11 (clone 3A4A7, Invitrogen, Carlsbad, UK), anti-PARP-1 (Cell Signaling Technology), anti-RPS6 (clone C-8, Santa Cruz Biotechnology, CA, USA), anti-RPS14 (clone H-130, Santa Cruz Biotechnology), anti-Mdm2 (clone SMP14 and clone H-221, Santa Cruz Biotechnology), anti-Slug (clone C19G7, Cell Signaling Technology), anti-E-cadherin (clone 24E10, Cell Signaling Technology), anti-c-Myc (clone N-262, Santa Cruz Biotechnology), anti-β-actin (clone AC-74, Sigma-Aldrich), and anti-Lamin B (C-20, Santa Cruz Biotechnology). Horseradish peroxidase-conjugated secondary antibodies were from GE-Healthcare (Milan, Italy).

### Cell invasion assay

NCM460 and HepG2 cells were seeded in the upper chamber in a serum-free medium in the presence or absence of IL-6 and/or aspirin 0.5 mM for 24 hours at 37°C. Invasion assay was performed as previously described [22].

### Evaluation of cell proliferation rate

NCM460 and HepG2 cell lines were treated with IL-6 and/or aspirin (from 0.1 to 3 mM) for 96 or 120 hours. Cell population was evaluated according to the Crystal Violet method as described previously [66].

### RNA extraction, reverse transcription and Real-Time RT-PCR

Total RNA was extracted with TRIreagent (Ambion, Austin, TX, USA), reverse-transcribed, and analyzed by Real-Time RT-PCR, as previously described [22]. Primers for SYBR Green Real-Time RT-PCR analysis of human 45S rRNA, RPS6, c-Myc, RPL11, RPS14, TP53, IL-6; mouse 45S rRNA and actin were designed using the Roche online primers design tool. β-glucuronidase mRNA was quantified with TaQMan Gene Expression Assays kit (Applied Biosystems). All sequences were reported in Supplementary Table S1.

### Assessment of RNA polymerase I activity

The NCM460 cell line was treated with 0.5 and 1.5 mM of aspirin for 24 h. Cell nuclei were extracted in a TKM buffer (10 mM Tris-HCl pH 7.4, 10 mM KCl, 3 mM MgCl2) and suspended in 10 mM Tris-HCl pH 7.4, 0.25 M sucrose, 1 mM MgCl2. RNA-Polymerase-I activity was assayed at high ionic strength in the presence of α-amanitin (Sigma-Aldrich). Five million nuclei of each cell line were incubated for 10 min at 37°C in 50 mM Tris-HCl pH 8, 0.2 mM MnCl2, 140 mM (NH4)2SO4, 0.9 mM ATP, GTP, CTP, 18 mM unlabeled UTP, 0.05 mM 3[H]UTP (PerkinElmer Italia, Milan, Italy), 1 μg/ml α-amanitin. Two volumes of trichloroacetic acid 5% were then added, and the radioactivity of the precipitated fraction was measured. Results were expressed in dpm/μg DNA.

### Capture of nascent 45s rRNA

NCM460 and HepG2 cell lines were treated with aspirin (1.5 mM) for 24 h. 5-EthynylUridine (EU, an alkyne-modified nucleoside) 0.1 mM was added into the culture medium and incorporated into the cells for 2 hours for the pulse condition. Then the medium was replaced with growth medium containing Uridine 0.2 mM for 2 hours for the chase condition. Total RNA was extracted. EU-labeled RNAs were biotinylated and captured by using the Click-it Nascent-RNA-Capture-Kit (Life Technologies, Eugene Oregon, USA), in accordance with the manufacturer's instructions, and used for cDNA synthesis.

### Quantitative evaluation of the 28S and 18S rRNA transcripts

NCM460 and HepG2 cell lines were treated with IL-6 and/or aspirin (0.5 or 1.5 mM) for 24 hours. Total RNA was extracted starting from the same cell number. The 28S and 18S RNA subunits were visualized by loading RNA in a 1% agarose gel stained with ethidium bromide. The intensity of bands was evaluated with the densitometric software GelPro analyzer 3.0 (Media Cybernetics, Silver Spring, MD).

### Polysome profile analysis

NCM460 cells were treated with 1.5 mM aspirin for 24 hours. At 70% of confluence, cells were treated with 100 μg/ml Cycloheximide (CHX) for 15 min. Cells were washed twice on ice in PBS + 100 μg/ml CHX, then resuspendend in a LSB buffer [20 mM Tris HCl, 10 μM NaCl, 3 mM MgCl2, 100 μg/ml CHX, 0.04 U/μl RiboLock RNAsi inhibitor (Thermo Scientific, Waltham, MA, USA) protease inhibitor cocktail (Roche Diagnostics, Basel, Switzerland)] and lysate on ice for 10 min adding a detergent buffer [0.3% Triton N101 50 mM Sucrose, 100 μg/ml CHX, 0.04 U/μl RiboLock RNAsi inhibitor (Thermo Scientific)]. The lysate was centrifuged at 14000 rcf for 10 min at 4°C. Ribosomes were then separated on chilled 15%–50% Sucrose Gradient (in LSB buffer by ultracentrifugation at 160000 × g for 2 hours at 4°C). The polysome profile was monitored at 254 nm (0.2 O.D. sensibility) and fractionated (at 10×, 10% Tris-Pump power) using an ISCO gradient fractionator system interfaced to an UA-6 absorbance detector (Teledyne Isco, Lincon, NE, USA). The data collected were digitally converted by using Minilab 1008 (Measuring Computing, Norton, MA, USA) and TracedDaq software (Measuring Computing, Norton, MA, USA), acquiring data in a differential mode at +/− 4 V and 4Hz.

### Gene silencing by RNAi transfection

Validated stealth RNAi siRNA (Invitrogen) targeting c-Myc, RPL11 and IL-6 were used as previously described [22]. Silenced control cells (scrambled) were transfected with equivalent amounts of Stealth RNAi negative control (Invitrogen). Cells were transfected with lipofectamine RNAiMAX (Invitrogen) in opti-MEM medium (Invitrogen), following the manufacturer's procedures.

### NOR silver staining

AgNOR staining and morphometric analysis were performed as described [67].

### Statistical analysis

The χ2 or Mann-Whitney *U* test, when appropriate, was used for the comparisons among groups. The agreement between scores was assessed by the κ statistics. All statistics were obtained using the SPSS statistical software package (SPSS, Inc.). *P* values < 0.05 were regarded as statistically significant.

## SUPPLEMENTARY MATERIALS FIGURES AND TABLE


