# Supplementary material for: Therapeutic dosages of aspirin counteract the IL-6 induced pro-tumorigenic effects by slowing down the ribosome biogenesis rate
Source: Oncotarget. 2016 Aug 20;7(39):63226–41. doi: 10.18632/oncotarget.11441 (PMC5325359; doi:10.18632/oncotarget.11441)
Supplement: Supplementary file 1 [file oncotarget-07-63226-s001.pdf]

Therapeutic dosages of aspirin counteract the IL-6 induced pro-tumorigenic effects by slowing down the ribosome biogenesis rate

Supplementary Materials

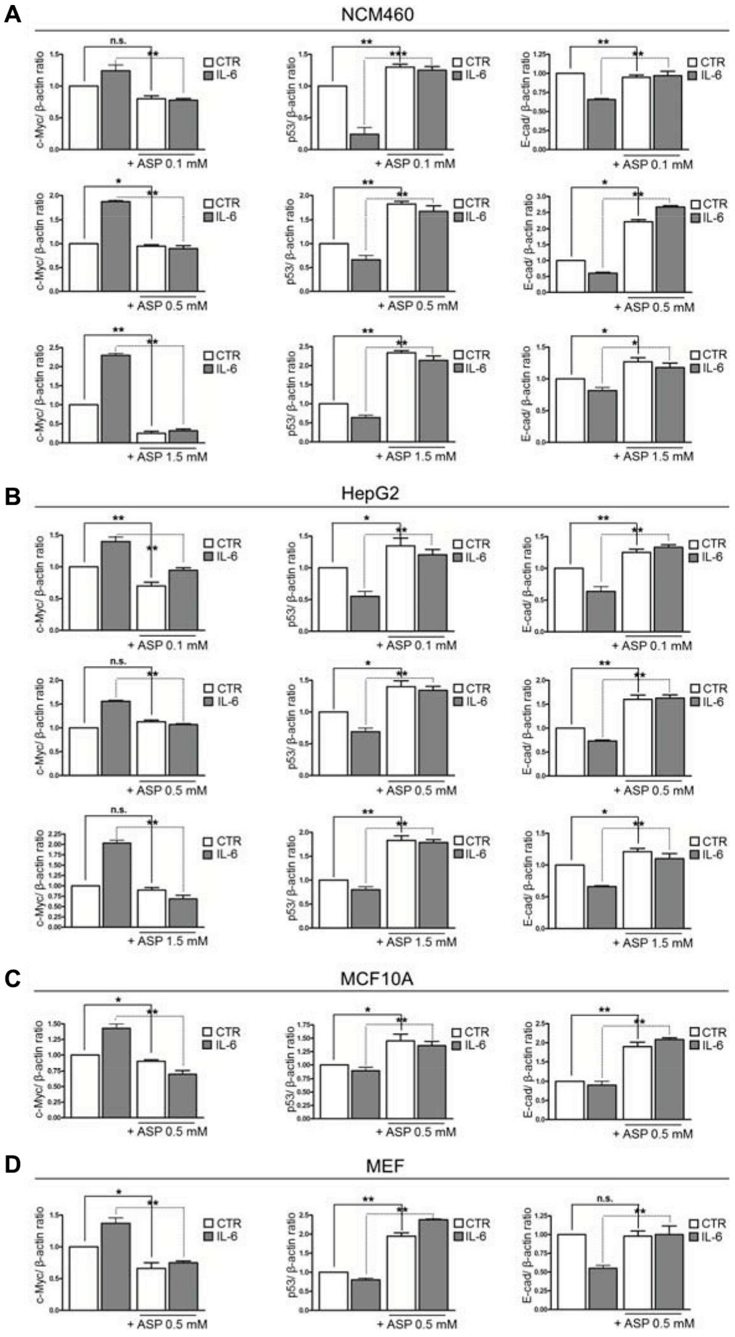

Supplementary Figure S1: Aspirin counteracts the effect of IL-6 on c-Myc, p53 expression and E-cadherin expression. Densitometric analysis of c-Myc, p53 and E-cadherin protein expression in control and IL-6-stimulated cells treated with aspirin for 24 hours. (**A**) NCM460 and HepG2 cells treated with aspirin at a concentration of 0.1 mM. (**B**) NCM460, HepG2, MCF10A and MEF cells treated with aspirin at a concentration of 0.5 mM. (**C**) NCM460 and HepG2 cells treated with aspirin at a concentration of 1.5 mM. The histograms show the values (mean ± s.d.) of three experiments. *$P < 0.05$; **$P < 0.01$. n.s., not significant.

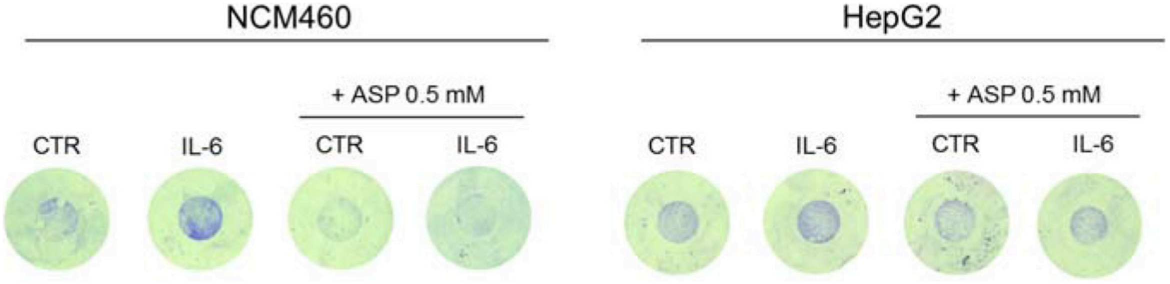

Supplementary Figure S2: Aspirin counteracts the effect of IL-6 on cell invasiveness. Invasion assay filters of NCM460 and HepG2 cells. Cells were exposed to IL-6 and/or to aspirin 0.5 mM for 24 hours. In both cell lines, aspirin counteracted the effect of IL-6 on the invasiveness potential.

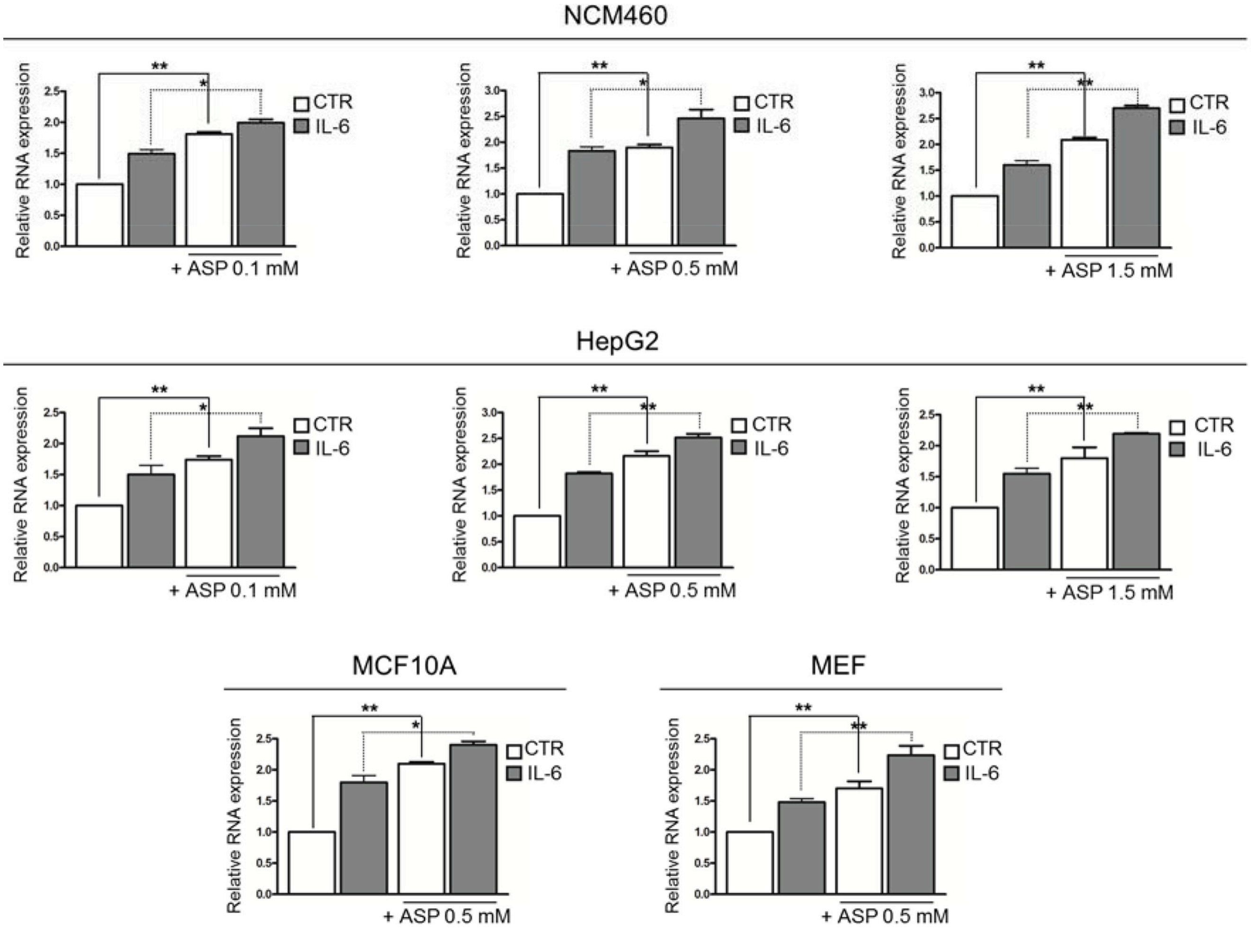

Supplementary Figure S3: Aspirin increases the 45S rRNA amount in control and IL-6-stimulated cells. Real-time RT-PCR analysis of the 45S rRNA expression in control and IL-6-stimulated NCM460, HepG2, MCF10A and MEF cells, treated with aspirin for 24 hours. The histograms show the values (mean ± s.d.) of three experiments. *$P < 0.05$; **$P < 0.01$.

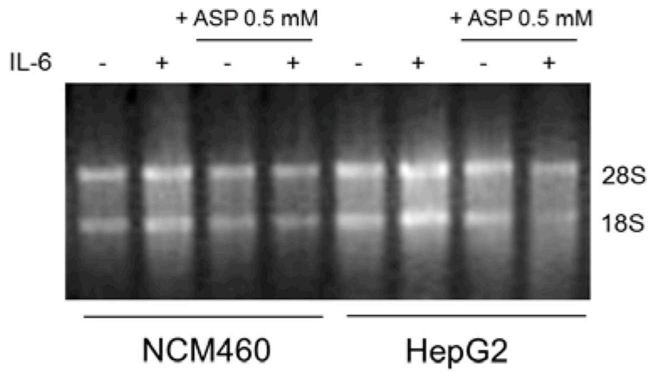

Supplementary Figure S4: : Aspirin counteracts the effect of IL-6 on 28S and 18S rRNA amount. NCM460 and HepG2 cells treated with 0.5 mM aspirin and/or IL-6 for 24 h. Total RNA was size-separated on 1% agarose gel and stained with ethidium bromide. Two bands corresponding to 28S and 18S rRNA are visible in each lane.

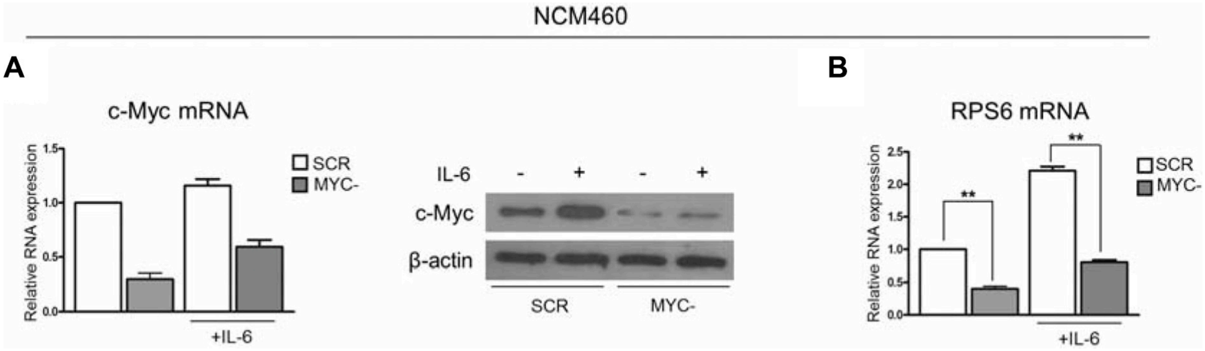

Supplementary Figure S5: c-MYC silencing and RPS6 mRNA expression in NCM460 cells. (**A**) Real-time RT-PCR evaluation of c-Myc mRNA expression and Western blot analysis of c-Myc protein expression in NCM460. The cells were interfered with scrambled (SCR) or c-Myc siRNA (MYC-) for 48 h. (**B**) Real-time PCR analysis of the RPS6 mRNA levels in NCM460 cells. The cells were interfered with scramble (SCR) or c-Myc siRNA (MYC-) for 48 h and then exposed to IL-6 for 24 hours. IL-6 greatly stimulated the RPS6 mRNA transcription; c-Myc interference caused a reduction in the RPS6 mRNA amount both in control and IL-6 stimulated cells. The histograms show the values (mean ± s.d.) of three experiments. $^{**}P < 0.01$.

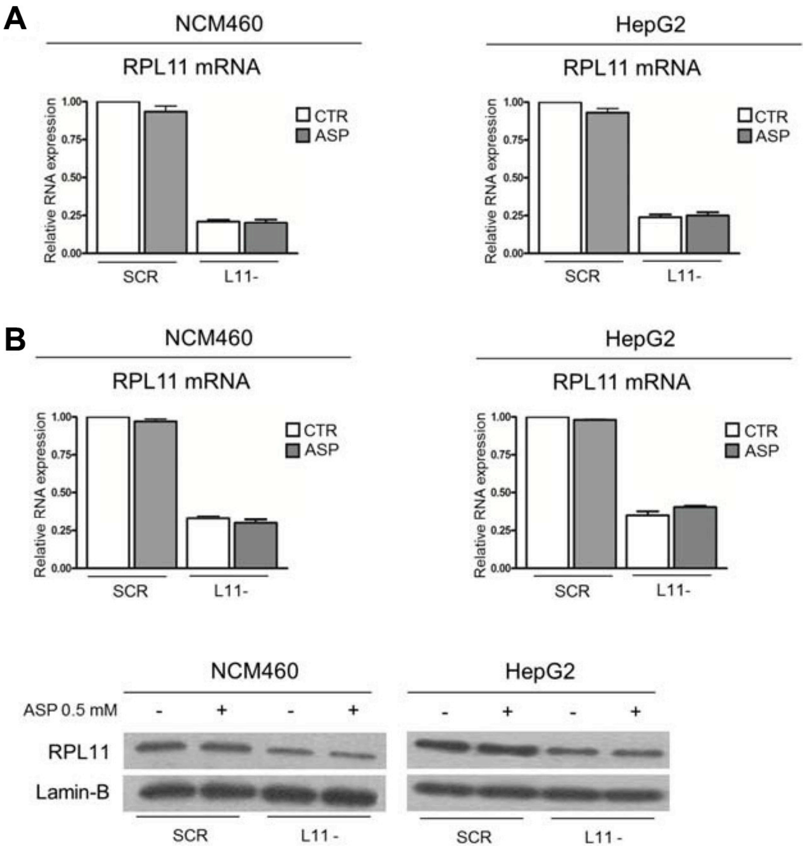

Supplementary Figure S6: Efficiency of RPL11 silencing. (**A**) siRNA1: Real-time RT-PCR evaluation of RPL11 mRNA expression in NCM460 and HepG2 cells. The cells were interfered with scrambled (SCR) or RPL11 siRNA1 (L11-) for 48 h. (**B**) siRNA2: Real-time RT-PCR evaluation of RPL11 mRNA expression and Western blot analysis of RPL11 protein expression in NCM460 and HepG2 cells. The cells were interfered with scrambled (SCR) or RPL11 siRNA2 (L11-) for 48h. The histograms show the values (mean ± s.d.) of three experiments.

NCM460

CTR

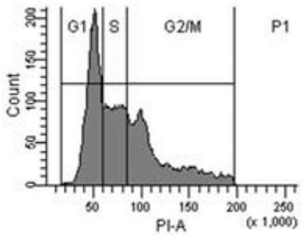

Population	#Events	%Parent	%Total
All Events	10,000		100.0
P1	8,985	89.8	89.8
G1	3,245	36.1	32.4
S	2,272	25.3	22.7
G2/M	3,468	38.6	34.7

ASP 0.5 mM

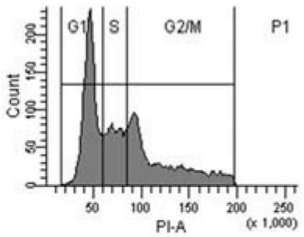

Population	#Events	%Parent	%Total
All Events	10,000		100.0
P1	8,483	84.8	84.8
G1	3,328	39.2	33.3
S	1,790	21.1	17.9
G2/M	3,365	39.7	33.7

ASP 1 mM

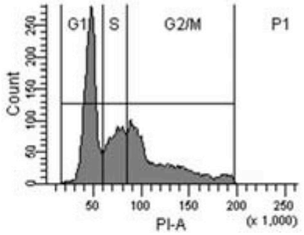

Population	#Events	%Parent	%Total
All Events	10,000		100.0
P1	8,982	89.8	89.8
G1	3,685	41.0	36.8
S	1,856	20.7	18.6
G2/M	3,441	38.3	34.4

ASP 1.5 mM

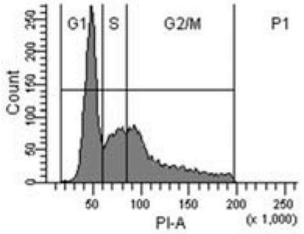

Population	#Events	%Parent	%Total
All Events	10,000		100.0
P1	8,916	89.2	89.2
G1	3,746	42.0	37.5
S	1,793	20.1	17.9
G2/M	3,377	37.9	33.8

ASP 3 mM

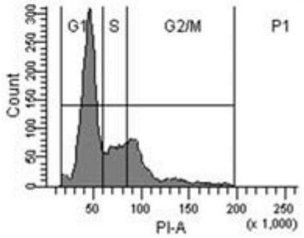

Population	#Events	%Parent	%Total
All Events	10,000		100.0
P1	8,969	89.7	89.7
G1	5,211	58.1	52.1
S	1,618	18.0	16.2
G2/M	2,140	23.9	21.4

Supplementary Figure S7: Cell cycle analysis. The NCM460 cell line was treated with different dosages of aspirin (0.5, 1, 1.5, 3 mM) for 24 h. Cells were collected and fixed in 70% ethanol at 4°C for 16 h. Fixed cells were then washed once with PBS, resuspended in 500 µl PBS containing 10 µg ml−1 propidium iodide and 50 µg ml−1 RNase and incubated for 30 min at room temperature. The cells were then centrifuged at 1200 rpm for 5 min, resuspended in 500 µl of PBS and analysed with Fluorescent-Activated Cell Sorter (BD FACSaria cell sorter, BD Biosciences, San Jose, CA, USA).

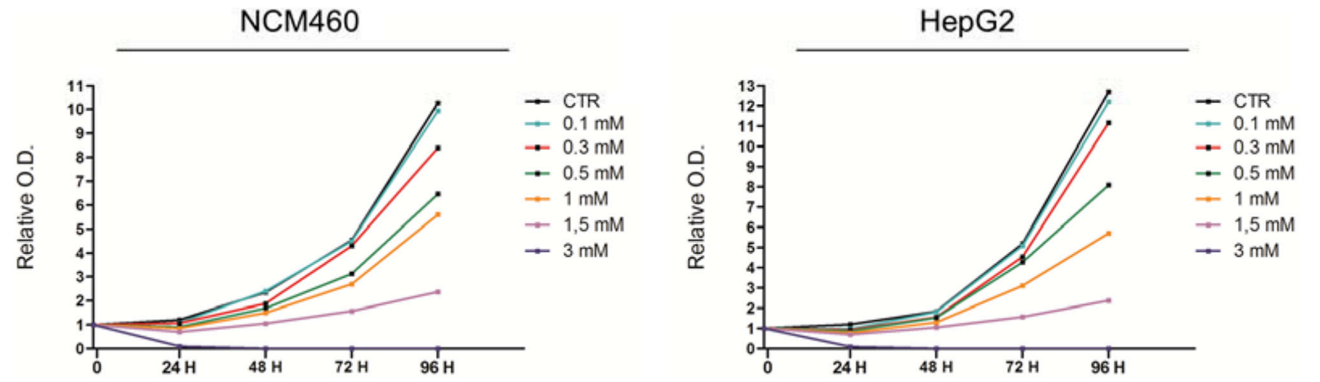

Supplementary Figure S8: Increasing aspirin concentrations induce a progressive reduction of the cell proliferation rate. Long-term effect (96 h) of aspirin treatment at the doses of 0.1, 0.3, 0.5, 1, 1.5 and 3 mM on the proliferation rate in NCM460 and HepG2 cells. O.D.: optical density. The IC50, calculated at 24 h, was 1.52 mM for NCM460 cells and 1.53 mM for HepG2 cells.

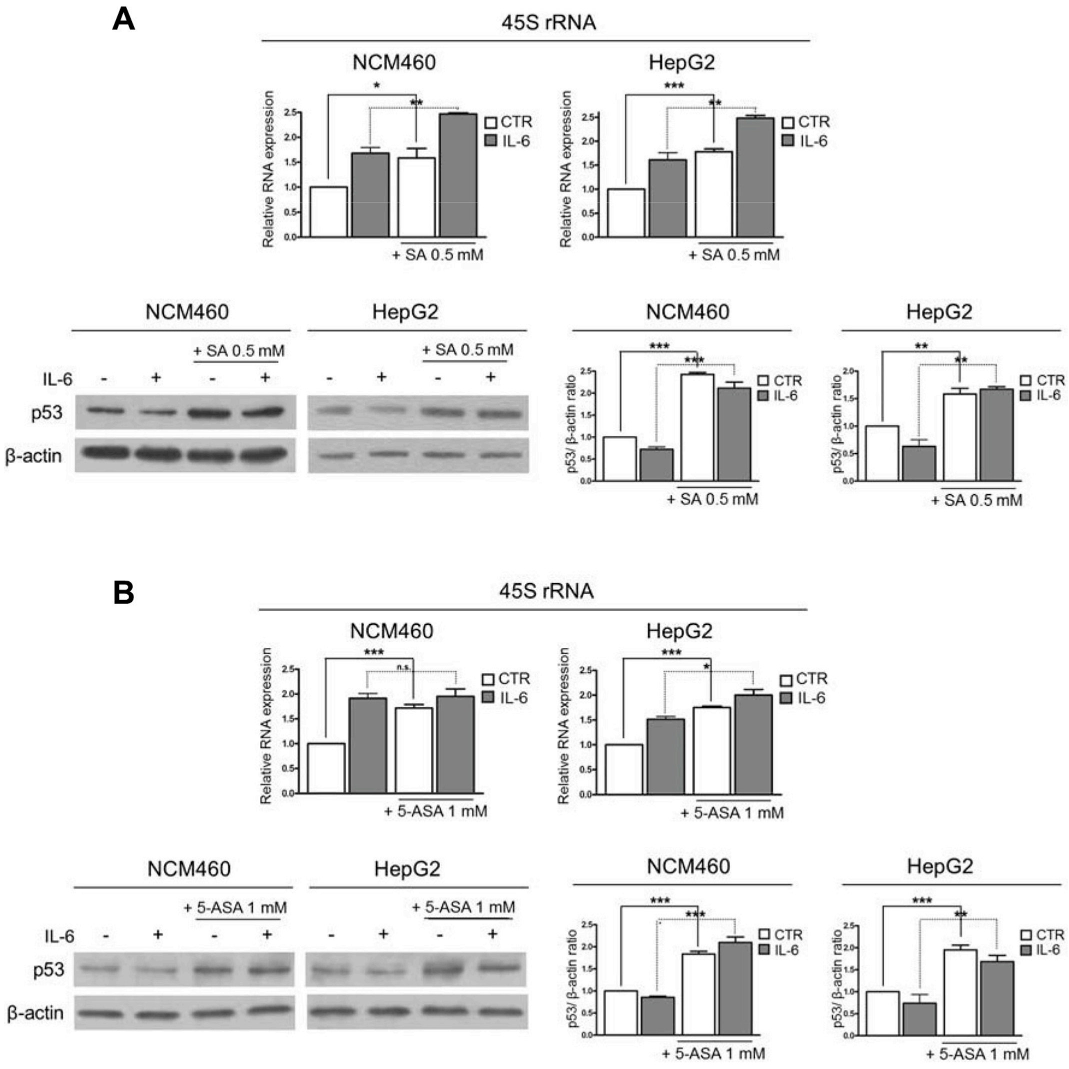

Supplementary Figure S9: IL-6 silencing and p53 expression in HepG2 cells. Real-time RT-PCR evaluation of 45S rRNA expression, Western blot and densitometric analysis of p53 in control and IL-6-stimulated NCM460 and HepG2 cells treated with (**A**) 0,5 mM salicylate (SA) or (**B**) 1 mM mesalazine (5-ASA) for 24 h. Both drugs counteract the effect of IL-6 on p53 expression. Moreover, drugs increase the 45S rRNA and p53 expression in control and IL-6-stimulated cells. The histograms show the values (mean ± s.d.) of three experiments. *$P < 0.05$; **$P < 0.01$; **$P < 0.001$.

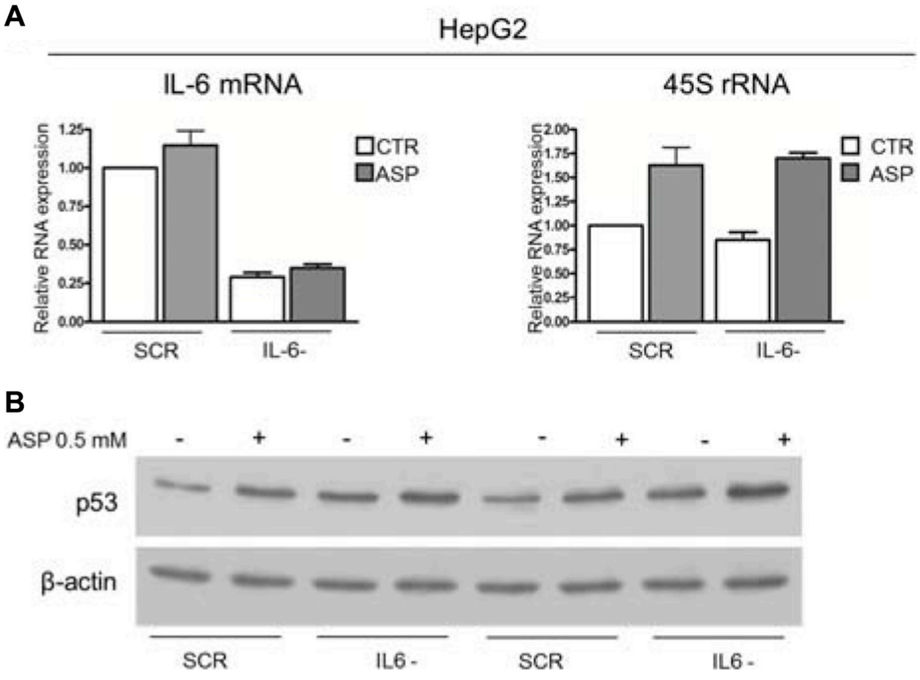

Supplementary Figure S10: IL-6 silencing and p53 expression in HepG2 cells. HepG2 cells were interfered with scramble (SCR) or IL-6 siRNA (IL-6-) for 48 h and then exposed to aspirin 0,5 mM for 24 hours. (**A**) Real-time RT-PCR evaluation of IL-6 mRNA and 45S rRNA expression in HepG2 cells. (**B**) Western blot analysis of p53 protein expression in HepG2 cells. Aspirin induced a p53 stabilization also in IL-6 silenced cells. The histograms show the values (mean ± s.d.) of three experiments.

Supplementary Table S1: Real-time RT-PCR primer sequences

Human primer sequences:

**45S rRNA**	FOR: 5′-GAACGGTGGTGTGTCGTT-3′<br>REV: 5′-GCGTCTCGTCTCGTCTCACT-3′
**c-Myc**	FOR: 5′-CTGCGACGAGGAGGAGGACT-3′<br>REV: 5′-GGCAGCAGCTCGAATTTCTT-3′
**RPS6**	FOR: 5′-TCTTGACCCATGGCCGTGTC-3′<br>REV: 5′-GCGGCAAGGCACTGTAGTAT-3′
**RPL11**	FOR: 5′-TCCACTGCACAGTTCGAGGG-3′<br>REV: 5′-AAACCTGGCCTACCCAGCAC-3′
**RPS14**	FOR: 5′-GCCTCGGACCTCAGGTGG-3′<br>REV: 5′-GTAGGGCGGTGATACCCAG-3′
**IL-6**	FOR: 5′-GAGAAAGGAGACATGTAACA-3′<br>REV: 5′-GCGCAGAATGAGATGAGTTGT-3′
**TP53**	FOR: 5′-TAACAGTTCCTGCATGGGC-3′<br>REV: 5′-AGGACAGGCACAAACACGC-3′

Mouse primer sequences:

**actin**	FOR: 5′-AGAGGGAAATCGTGCGTGAC-3′<br>REV: 5′-CAATAGTGATGACCTGGCCGT-3′
**45S rRNA**	FOR: 5′-CTCCTGTCTGTGGTGTCCAA-3′<br>REV: 5′-TGATACGGGCAGACACAGAA-3′
